# CCR5 Receptor Occupancy Analysis Reveals Increased Peripheral Blood CCR5+CD4+ T Cells Following Treatment With the Anti-CCR5 Antibody Leronlimab

**DOI:** 10.3389/fimmu.2021.794638

**Published:** 2021-11-19

**Authors:** Xiao L. Chang, Helen L. Wu, Gabriela M. Webb, Meenakshi Tiwary, Colette Hughes, Jason S. Reed, Joseph Hwang, Courtney Waytashek, Carla Boyle, Cleiton Pessoa, Andrew W. Sylwester, David Morrow, Karina Belica, Miranda Fischer, Scott Kelly, Nader Pourhassan, Rachele M. Bochart, Jeremy Smedley, Christopher P. Recknor, Scott G. Hansen, Jonah B. Sacha

**Affiliations:** ^1^ Vaccine and Gene Therapy Institute, Oregon Health and Science University, Portland, OR, United States; ^2^ Oregon National Primate Research Center, Oregon Health and Science University, Portland, OR, United States; ^3^ CytoDyn Inc., Vancouver, WA, United States

**Keywords:** CCR5, CD4, HIV, receptor occupancy (RO), flow cytometry, antibody

## Abstract

CCR5 plays a central role in infectious disease, host defense, and cancer progression, thereby making it an ideal target for therapeutic development. Notably, CCR5 is the major HIV entry co-receptor, where its surface density correlates with HIV plasma viremia. The level of CCR5 receptor occupancy (RO) achieved by a CCR5-targeting therapeutic is therefore a critical predictor of its efficacy. However, current methods to measure CCR5 RO lack sensitivity, resulting in high background and overcalculation. Here, we report on two independent, flow cytometric methods of calculating CCR5 RO using the anti-CCR5 antibody, Leronlimab. We show that both methods led to comparable CCR5 RO values, with low background on untreated CCR5+CD4+ T cells and sensitive measurements of occupancy on both blood and tissue-resident CD4+ T cells that correlated longitudinally with plasma concentrations in Leronlimab-treated macaques. Using these assays, we found that Leronlimab stabilized cell surface CCR5, leading to an increase in the levels of circulating and tissue-resident CCR5+CD4+ T cells *in vivo* in Leronlimab-treated macaques. Weekly Leronlimab treatment in a chronically SIV-infected macaque led to increased CCR5+CD4+ T cells levels and fully suppressed plasma viremia, both concomitant with full CCR5 RO on peripheral blood CD4+ T cells, demonstrating that CCR5+CD4+ T cells were protected from viral replication by Leronlimab binding. Finally, we extended these results to Leronlimab-treated humans and found that weekly 700 mg Leronlimab led to complete CCR5 RO on peripheral blood CD4+ T cells and a statistically significant increase in CCR5+CD4+ T cells in peripheral blood. Collectively, these results establish two RO calculation methods for longitudinal monitoring of anti-CCR5 therapeutic antibody blockade efficacy in both macaques and humans, demonstrate that CCR5+CD4+ T cell levels temporarily increase with Leronlimab treatment, and facilitate future detailed investigations into the immunological impacts of CCR5 inhibition in multiple pathophysiological processes.

## Introduction

C-C chemokine receptor type 5 (CCR5) is a G protein-coupled receptor involved in cell signaling and migration. CCR5 is primarily expressed in lymphocytes, macrophages, dendritic cells, and natural killer cells, but can also be found on the epithelium, endothelium, vascular smooth muscle, and fibroblasts from multiple organs, as well as neurons, astrocytes, and microglia in the central nervous system (CNS) (1, 2). Chemokines CCL3 (MIP-1α), CCL4 (MIP-1β), and CCL5 (RANTES) are the primary CCR5 ligands and are expressed in sites of inflammation to recruit CCR5+ immune cells; thus, the number of CCR5+ cells often correlate with the severity of inflammation (1, 3, 4).

While CCR5 is known for its role in facilitating human immunodeficiency virus (HIV) infection of CD4+ T cells, it has a wide range of roles in normal and pathophysiological processes. In addition to HIV, CCR5 is a critical host receptor for Dengue virus (5) and Staphylococcus aureus (6) infection. Furthermore, high expression of CCR5 is associated with cancer progression and tumorigenesis (7–9), development of insulin resistance *via* adipose tissue macrophage recruitment (10), and suppression of cortical plasticity, learning, and memory (11–13). Moreover, individuals homozygous for the naturally occurring CCR5 mutation, CCR5Δ32, lack cell surface expression of CCR5 receptors, which has protective effects against HIV infection (14, 15), asthma (16, 17), severe SARS-CoV-2 symptoms (18), and development of rheumatoid arthritis (19), and is associated with improved hepatitis B virus infection recovery rates (20) and lower incidence of cardiovascular disease (21, 22). However, CCR5 is critical for viral clearance after infection by West Nile (23), Japanese encephalitis (24), and influenza A viruses (25, 26) because of its role in trafficking immune cells to sites of infection. Exemplifying the complexity of CCR5, the lack of CCR5 receptors protected against parasitic *Toxoplasma gondii* infection (27) while the presence of CCR5 was essential for disease control after infection (28, 29). Because of the myriad roles played by CCR5, the ability to target CCR5 with therapeutic agents will have a diverse range of applications.

Widely used HIV-specific therapeutics, such as antiretroviral (ARV) drugs and HIV-specific broadly neutralizing antibodies (bNAbs), exert their antiviral effects by directly targeting viral proteins, and thus their efficacy is correlated to plasma concentration (30–32). However, for immunomodulatory and antiviral agents that target host receptors, their efficacy relies on the level of receptor occupancy (RO) achieved. Indeed, in HIV infection the density of CCR5 on CD4+ T cells correlated closely to both susceptibility to HIV infection *in vitro* and plasma viral loads in HIV-infected individuals (33–35). Additionally, the paucity of CCR5+ CD4+ T cells present in natural hosts of simian immunodeficiency virus (SIV) during infancy protects against mother-to-offspring transmission during breastfeeding by viremic mothers (36). Thus, the level of CCR5 occupied by a CCR5-targeting drug is a critical predictor of its therapeutic efficacy.

Currently, Maraviroc is the only FDA-approved CCR5 antagonist. Maraviroc inhibits CCR5 internalization following ligand binding, and thus Maraviroc CCR5 RO is indirectly measured by a MIP-1β internalization assay where CCR5 RO is defined by the percentage of cell surface CCR5 that is not down-regulated following treatment with MIP-1β (37). This indirect method of measuring CCR5 RO results in background RO of approximately 25% (38), with reports of 120% CCR5 RO in peripheral blood CD4+ T cells from both Maraviroc-treated and -untreated rhesus macaques (39). These issues of extremely high background and overcalculation of CCR5 RO are major limitations of the MIP-1β internalization assay, especially when CCR5 RO is a critical parameter in studying CCR5-blocking agents.

Directly measuring CCR5 RO with monoclonal antibodies also presents challenges as CCR5 expression is a dynamic process that must be controlled for. Indeed, the frequency of CCR5+ cells change longitudinally in response to inflammatory and homeostatic stimuli and can be impacted by the CCR5-targeting reagent itself (39–43), leading to inaccuracies in methods that use baseline CCR5 values to calculate CCR5 RO (44). Not accounting for the ability of CCR5 expression to change over time in the CCR5 RO calculation for the anti-CCR5 antibody HGS004 resulted in baseline pre-treatment CCR5 RO values of 20% in HIV-1 infected participants (45). Thus, no robust and highly sensitive method for the calculation of CCR5 RO currently exists.

Here, we report on two sensitive methods to measure CCR5 RO by the anti-CCR5 antibody Leronlimab (PRO-140; Vyrologix). We demonstrate the sensitivity of this method to longitudinally quantify CCR5 RO on blood and tissue CD4+ T cells from Leronlimab-treated macaques and describe increased levels of CCR5+CD4+ T cells in the blood of both Leronlimab-treated macaques and humans. Finally, we translate the macaque CCR5 RO method to Leronlimab-treated, HIV-naïve human participants, demonstrating the direct use for monitoring CCR5 RO by Leronlimab in human clinical trials.

## Materials and Methods

### Study Approval and Design (Non-Human Primates)

All animal work occurred at the Oregon National Primate Research Center (ONPRC), a Category I facility that is fully credited by the American Association for Accreditation of Laboratory Animal Care (AAALAC), with approved Assurance (#A3304-01) for the use and care of animals on file with the NIH Office of Laboratory Animal Welfare. Animal experimental care plans, protocols, procedures, and administered reagents were approved by ONPRC Institutional Animal Care and Use Committee (IACUC). The ONPRC IACUC adhers to the national guidelines established in the Animal Welfare Act (7 U.S.C. Sections 2131–2159) and the Guide for the Care and Use of Laboratory Animals (8th Edition) as mandated by the U.S. Public Health Service Policy.

Macaques (*Macaca mulatta and Macaca fascicularis*) used in this study were housed at the Oregon National Primate Research Center (ONPRC) in Animal Biosafety level (ABSL)-2+ rooms with autonomously controlled temperature, humidity, and lighting. At assignment, macaques were free of Cercopithecine herpesvirus 1, D-type simian retrovirus, simian T-lymphotropic virus type 1, and Mycobacterium tuberculosis. Macaque specialists designed and oversaw daily wellness and dietary enrichment plans. Ketamine HCl (Ketathesia™, Henry Schein Animal Health) with or without Dexmedetomidine (Dexmedesed™, Dechra, Overland Park, KS) was used to sedate macaques for procedures, including subcutaneous (SC) injections of Leronlimab, venipuncture, tissue biopsy, and viral challenge, and they were performed by certified veterinarians or trained animal technicians with veterinarian oversight.

Adult rhesus macaques (n=12) were used in this study, with six macaques in the single 10 mg/kg SC Leronlimab group and six macaques in the single 50 mg/kg SC Leronlimab group. All but one animal in the 10 mg/kg group were female. Baseline biopsies and whole blood were collected before SC Leronlimab injections. Blood was collected at eight, 24, 48, and 72 hours post Leronlimab injection, and then weekly afterwards. Biopsies were collected at one and four weeks after Leronlimab injection and processed as described below. This was a non-terminal study and macaques were returned to the ONPRC colony after study conclusion.

One adult female chronically SIVmac239-infected Mauritian cynomolgus macaque received weekly doses of 50 mg/kg Leronlimab subcutaneously for 11 weeks.

### Study Approval and Design (Human)

De-identified peripheral blood samples were obtained from participants in a phase 2, randomized, double blind, placebo-controlled study to evaluate for the efficacy and safety of Leronlimab treatment in human participants experiencing prolonged SARS-CoV-2 symptoms (termed Long-Haulers). Participants were randomized to receive weekly 700 mg SC Leronlimab or placebo. Participants of all sexes, over 18 years of age, and with prior confirmed positive SARS-CoV-2 RT-PCR test were eligible. Participants experienced at least two clinical symptoms consistent with a SARS-CoV-2 infection for more than 12 weeks. The trial took placed at the Arthritis & Rheumatic Disease Specialties in Aventura, Florida and Center for Advanced Research & Education (CARE) in Gainesville, Georgia under the ClinicalTrials.gov identifier (NCT number): NCT04678830. All participants provided written informed consent prior to inclusion in study.

### Tissue Processing

Whole blood, lymph node, and bone marrow samples were processed as previously described (40). Briefly, peripheral blood mononuclear cells (PBMCs) were isolated from whole blood by density gradient centrifugation using Ficoll-Paque PLUS (Cytiva) and centrifuged at 1,860 x g for 30 minutes. Plasma was saved for viral nucleic acid and Leronlimab concentration detection. Buffy coat containing PBMCs was collected and washed with R10 (RPMI 1640 containing 10% fetal bovine serum (FBS)) before use. Lymph nodes were collected in R10, diced with a scalpel, and forced through a 70 μm cell strainer to a single cell suspension in R10. Bone marrow aspirates were collected in R10 and pelleted by centrifugation at 830 x g for 4 minutes. Cell pellets were resuspended by shaking in PBS containing 2 mM EDTA, and then centrifuged at 830 x g for 4 minutes. Cell pellets were then resuspended in 70% isotonic Percoll (GE Healthcare, Buckinghamshire, UK) and layered under 37% isotonic Percoll. Layers were centrifuged at 500 x g for 20 minutes. Mononuclear cells in the interface were collected into R10.

### CCR5 RO Assay

#### Equation 1 Flow Staining

Equation 1 measured for Leronlimab-occupied CCR5 by using anti-IgG4 FITC in comparison to total CCR5 measured by *ex vivo* incubation with a saturating concentration of Leronlimab (5 μg/mL). Three staining tubes were used, with one tube serving as the fluorescence minus one (FMO) control (**Supplemental Table 1**). A minimum of 50 μL of whole blood or 3 x 10^5^ mononuclear cells were used per staining tube. Cells were washed twice with 1mL of PBS by centrifuging at 830 x g for 4 minutes, then supernatants were aspirated to leave ~100 μL, and finally cell pellets were resuspended by vortex. Next, 5 μg/mL of parental, unconjugated Leronlimab was added to tube 3 according to **Supplemental Table 1** and incubated for 30 minutes at room temperature in the dark. Cells were washed once with PBS, and then anti-IgG4 FITC was added to tubes 2 and 3 and incubated for 30 minutes at room temperature in the dark. Afterwards, cells were washed once with PBS + 10% FBS and once with PBS. Anti-CCR5 APC was added to tubes 2 and 3, while surface antibodies (CD3, CD4, CD8, CD45, CD95, and CD28) and amine-reactive dye (cell viability) were added to all tubes. A description of antibodies used can be found in **Supplemental Table 2**. Cells were incubated for 30 minutes at room temperature in the dark. For whole blood samples, 1 mL of 1X FACS Lysing solution (BD Biosciences) was added to each tube to lyse red blood cells for 8 minutes, and then immediately centrifuged and washed three times with PBS + 10% FBS. Cells were stored at 4°C prior to running on BD™ LSR II flow cytometer (BD Biosciences). For mononuclear cells, cells were washed twice with PBS and then fixed by adding 100 μL of 2% paraformaldehyde (PFA) and incubating for at least 10 minutes before running on BD™ LSR II flow cytometer (BD Biosciences). Flow analysis was done using FlowJo 10.4, where cells were progressively gated on singlets (FSC-H vs. FSC-A), live, CD45+, CD3+, CD4+/CD8-, and CCR5+ cell populations, as described in **Supplemental Figure 1**. Staining tube 1 served as the FMO control to assist with gating on desired cell populations, tube 2 stained for the frequency of Leronlimab-occupied CCR5+CD4+ T cells, and tube 3 was saturated with Leronlimab *ex vivo* to measure for the total frequency of CCR5+CD4+ T cells. The equation to calculate CCR5 RO using equation 1 is as follows:


$$\%RO=\frac{\%IgG4\;(tube\;2)}{\%IgG4\;(tube\;3)}\times 100\%$$

#### Equation 2 Flow Staining

Equation 2 measured for unoccupied CCR5 by using Leronlimab that was conjugated to Pacific Blue (termed Leronlimab-PB). Three staining tubes were required, with two tubes serving as the FMO controls (**Supplemental Table 1**). Similar to equation 1, a minimum of 50 μL whole blood or 3 x 10^5^ mononuclear cells were used per staining tube. Cells were washed two times with PBS and anti-IgG4 FITC was added to tube C following **Supplemental Table 1** and incubated for 30 minutes at room temperature in the dark. Because anti-IgG4 FITC could interact with downstream Leronlimab-PB, leading to false positive staining of anti-IgG4 FITC, cells were washed once with PBS + 10% FBS and at least three times with PBS. Afterward, anti-CCR5 APC was added to tubes B and C, Leronlimab-PB was added to tube C, surface antibodies (CD3, CD4, CD8, CD45, CD16, and CD14) and amine-reactive dye were added to all tubes (**Supplemental Table 2**). Cells were incubated for 30 minutes at room temperature in the dark. Afterward, whole blood or mononuclear cells were lysed with 1X FACS Lysing solution or fixed with 2% PFA, respectively, as described in the staining for equation 1. Samples were collected on a BD™ LSR II flow cytometer (BD Biosciences) and analyzed with FlowJo 10.4, where cells were progressively gated on CD45+, singlets (FSC-H vs. FSC-A), live, CD3+, CD4+/CD8-, and CCR5+ cell populations (**Supplemental Figure 1**). Here, staining tube A served as a FMO control to assist with gating on desired cell populations, and tube B served as a FMO control to assist with gating on IgG4+ and Leronlimab-PB+ cell populations. Tube C stained for cells with unoccupied CCR5 (Leronlimab-PB) and occupied CCR5 (anti-IgG4 FITC), with the total frequency of CCR5+CD4+ T cells represented by the sum of IgG4+ and Leronlimab-PB+ cell frequencies. The equation to calculate CCR5 RO using equation 2 is as follows:


$$\%RO=\frac{\%IgG4\;(tube\;C)}{\%IgG4\;(tube\;C)+\%Leronlimab–PB\;(tube\;C)}\times 100\%$$

Lastly, the percentage of CCR5+CD4+ T cells was determined with tube B because it was free from *ex vivo* incubation of unconjugated Leronlimab or conjugated Leronlimab-PB, which, described later in the Results session, was found to stabilize and increase CCR5 expression on CD4+ T cells.

#### Combined Equations 1 and 2 Flow Staining

In the clinically-adapted CCR5 RO assay, changes were made to 1) fluorophore conjugates to adjust for the higher-wattage lasers used in FACSymphony A5 (BD Biosciences), a machine that allowed for easier detection of rare events, 2) the FACSymphony A5’s lack of a 651nm (green) laser and expanded 488nm (blue) laser, 3) antibody clones to improve species reactivity to macaque and human samples, and 4) combine both CCR5 RO equations into one staining panel to minimize the number of cells required for staining. Here, four staining tubes were required, with two tubes serving as FMO controls (**Supplemental Table 1**). At least 50 μL of whole blood or 3 x 10^5^ PBMC samples were placed into each staining tube and samples were washed two times with PBS. Next, 5 μg/mL of the parental, unconjugated Leronlimab was added to tube H4 (**Supplemental Table 1**) and incubated for 30 minutes at 2-8°C in the dark. Afterward, tubes H1 and H2 were washed once with 3 mL cold PBS while tubes H3 and H4 were washed with 1 mL cold PBS with 10% mouse serum (Equitech) in order to minimize nonspecific binding of anti-human IgG4 FITC. Cells in tubes H3 and H4 were then incubated with 100 μL of cold mouse serum and incubated for 60 minutes at 2-8°C followed by another wash of 1 mL cold PBS with 10% mouse serum. Anti-IgG4 FITC and 100 μL of cold mouse serum were then added to tubes H3 and H4 and incubated for 30 minutes at 2-8°C in the dark. Next, cells were washed once with DPBS and 0.1% BSA and then at least three times with PBS. Anti-CCR5 APC was added to tubes H2, H3, and H4, Leronlimab-PB was added to tube H3, surface antibodies (CD3, CD4, CD8a, CD45, CD16, CD14, CD28, and CD95), and amine-reactive dye (cell viability) were added to all tubes (**Supplemental Table 3**). Cells were incubated for 30 minutes at room temperature in the dark. Finally, whole blood or PBMC samples were lysed with 1X FACS Lysing solution, incubated for 10 minutes in the dark, and then immediately washed once with DPBS and 0.1% BSA. Cells were then permeabilized with 0.5 mL 1.5X FACS Lysing solution and 0.05% Tween-20, incubated for 10 minutes in the dark, and then washed twice with DPBS and 0.1% BSA. Samples were run using the FACSymphony A5 (BD Biosciences) and analyzed with FlowJo 10.4. Cells were progressively gated on CD45+, singlets (FSC-H vs. FSC-A), live, CD3+, CD4+/CD8-, and CCR5+ cell populations (**Supplemental Figure 1**). Here, tube H1 and H2 served as FMO controls to assist with gating on cell populations, tube H3 stained for the frequency of Leronlimab-occupied CCR5 with anti-IgG4 FITC and Leronlimab-unoccupied CCR5 with Leronlimab-PB, and tube H4 was saturated with Leronlimab *ex vivo* to measure for the total frequency of CCR5+CD4+ T cells. The equations to calculate CCR5 RO are as follow:


Equation 1 $$\%RO=\frac{\%IgG4\;(tube\;H3)}{\%IgG4\;(tube\;H4)}\times 100\%$$

Equation 2 $$\%RO=\frac{\%IgG4\;(tube\;H3)}{\%IgG4\;(tube\;H3)+\%\mathrm{Leronlimab}–PB\;(tube\;H3)}\times 100\%$$

### *In Vitro* CCR5 Ligand Binding and Staining

PBMCs were incubated with or without 5 μg/mL of Leronlimab for 30 minutes at 37°C. Next, cells were incubated at 37°C for an additional 30 minutes with no additional ligand or 50 nM of one of the following CCR5 ligands: MIP-1α, MIP-1β, or RANTES. Afterwards, cells were stained with CCR5 (clone 3A9), CD3, CD4, and CD8 cell surface receptors and amine-reactive dye. Samples were collected using the BD™ LSR II flow cytometer and analyzed with FlowJo 10.4 by gating on live, CD3+, CD4+/CD8-, singlet, and CCR5+ cell populations. Data was presented as normalized %CCR5+ for each treatment condition that was normalized to frequency observed in Leronlimab-untreated, ligand-free cells from each respective donor.

### Measurement of Leronlimab Concentration in Plasma

Enzyme-linked immunosorbent assay (ELISA) was used to detect for Leronlimab levels in plasma samples, as previously described (40). Briefly, plates were coated with 1.5 μg/mL PA22 (CytoDyn, Vancouver, WA) in carbonate-bicarbonate buffer (ThermoFisher) overnight at 4°C. Plates were washed three times with PBS-T (PBS with 0.1% Tween-20) and blocked for at least two hours in room temperature with Blocking Buffer (PBS with 0.4% Tween-20 and 10% bovine serum albumin (Fisher Scientific). Standard curve generated with serial dilutions of Leronlimab and samples were plated onto blocked plates and incubated for 30 minutes at room temperature. Plates were washed three times with PBS containing 0.5 M NaCl, and then incubated with 20,000-fold diluted mouse anti-human IgG4 pFc’-horseradish peroxidase (Southern Biotech) in Blocking Buffer for 30 minutes at room temperature. Finally, plates were washed three times with PBS-T and 3,3’,5,5’-Tetramethylbenzidine (TMB) substrate (Southern Biotech) was added to develop the plates for two minutes, after which, 1 N H2SO4 was added to stop the reaction. Developed plates were read on Synergy HTX Multi-Mode Microplate Reader (BioTek) using the Gen5 v3.10 software to read two absorbance wavelengths: 650 nm for the developing reaction and 450 nm for the developed reaction. Plasma concentration for each sample (μg/mL) was determined using the generated standard curve, with an assay limit of detection of 0.0226 μg/mL.

### Viral Nucleic Acid Detection

SIV nucleic acid detection assays were performed by members of the ONPRC Molecular Virology Core. Detection of SIV nucleic acids was performed as previously described (40, 46, 47). Briefly, viral nucleic acids from 300 μL of plasma were extracted using LEV Viral Nucleic Acid Kit and the Maxwell 16 instrument (Promega, Madison, WI) following manufacturer’s protocols. RT-qPCR reaction was performed to quantify SIV viral RNA in the plasma. The reaction used the total extracted RNA, 900 nM of SGAG21 forward primer (GTCTGCGTCATPTGGTGCATTC), 900 nM of SGAG22 reverse primer (CACTAGKTGTCTCTGCACTATPTGTTTTG), and 250 nM of pSGAG23 probe (5′-6-carboxyfluorescein [FAM]-CTTCPTCAGTKTGTTTCACTTTCTCTTCTGCG-black hole quencher [BHQ1]-3′) for a final reaction volume of 30 μL. Standard curve was created by using *in vitro* transcribed SIVgag RNA that was serially diluted in 5 ng/μL yeast tRNA (Sigma R5636). Applied Biosystems QuantStudio 6 Flex instrument (Life Technologies) was used to run the RT-qPCR reactions at the following setting: 50°C for 5 min; 95°C for 20 s; [95°C for 3 s, 60°C for 30 s] × 45 cycles. The limit of quantification for this assay is 50 copies/mL.

### Statistical Analysis

Wilcoxon signed-rank test was used to analyze %CCR5+CD4+ T cells between baseline and Leronlimab treatment timepoints in macaques. Two-way repeated measures analysis of variance (ANOVA) with Sidak’s correction for multiple comparisons was used to compare the %CCR5+CD4+ T cells between Leronlimab-treated and placebo human participants. Analysis was performed using GraphPad Prism Version 8.31 (332).

## Results

### Development of the Leronlimab CCR5 Receptor Occupancy Assay

Rhesus macaques are vitally important pre-clinical models used to validate potential prophylactic and therapeutic modalities as they share similar immune systems with humans. Indeed, CCR5 sequence, structure, and function are highly conserved between the two species (48). Previously, we demonstrated that Leronlimab specifically binds the same CCR5 epitope on the surface of human and rhesus macaque leukocytes (40). Therefore, to facilitate both pre-clinical macaque and clinical human studies, we analyzed the effect of Leronlimab on CCR5 expression on CD4+ T cells from both species.

**Figure 1:**
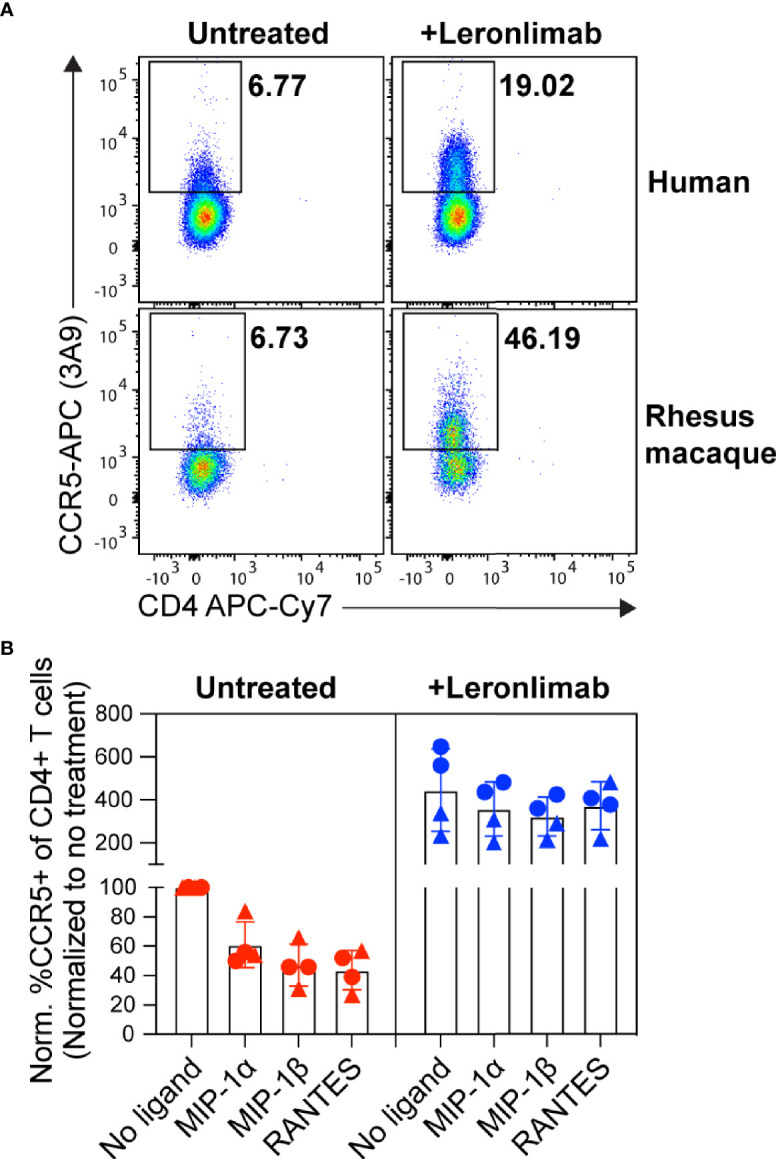
Leronlimab increases cell surface CCR5 expression that is resistant to internalization. PBMC from humans (n=2; triangles) and rhesus macaques (n=2; circles) were incubated without (left, red) or with (right, blue) 5 μg/mL Leronlimab for 30 minutes at 37°C, then with either no treatment or 50nM of one the following CCR5 ligands: MIP-1α, MIP-1β, or RANTES, for an additional 30 minutes. After incubation, cells were stained for CCR5 (clone 3A9) and CD3, CD4, and CD8 surface markers. Cells were gated within live, CD3+, CD4+/CD8-, singlet populations. **(A)** Representative flow plots. **(B)** Graphs show normalized frequencies of CCR5+ of CD4+ T cells to frequency observed with untreated cells (no Leronlimab, no CCR5 ligands) from the respective donor.

First, we examined the impact of treatment with a saturating concentration of 5 μg/mL Leronlimab on surface CCR5 levels of primary human and macaque CD4+ T cells *in vitro.* Similar to Maraviroc, Leronlimab stabilized cell surface CCR5 and directly increased the frequency of CCR5+CD4+ T cells from humans and rhesus macaques (**Figure 1A**). Next, we explored if Leronlimab-occupied CCR5 was resistant to internalization following treatment with CCR5 ligands, a phenomenon that forms the basis for the MIP-1β internalization Maraviroc CCR5 RO assay (37, 38). In samples without Leronlimab, we observed CCR5 internalization in response to MIP-1α, MIP-1β, and RANTES (**Figure 1B**). Following treatment with Leronlimab, we found increased frequencies of CCR5+CD4+ T cells where CCR5 was resistant to internalization following treatment with all three CCR5 ligands, indicating that Leronlimab both stabilized surface CCR5 expression and prevented its internalization. Thus, it is critical to account for this Leronlimab-induced increase in surface CCR5 levels for CCR5 RO measurements.

**Figure 2:**
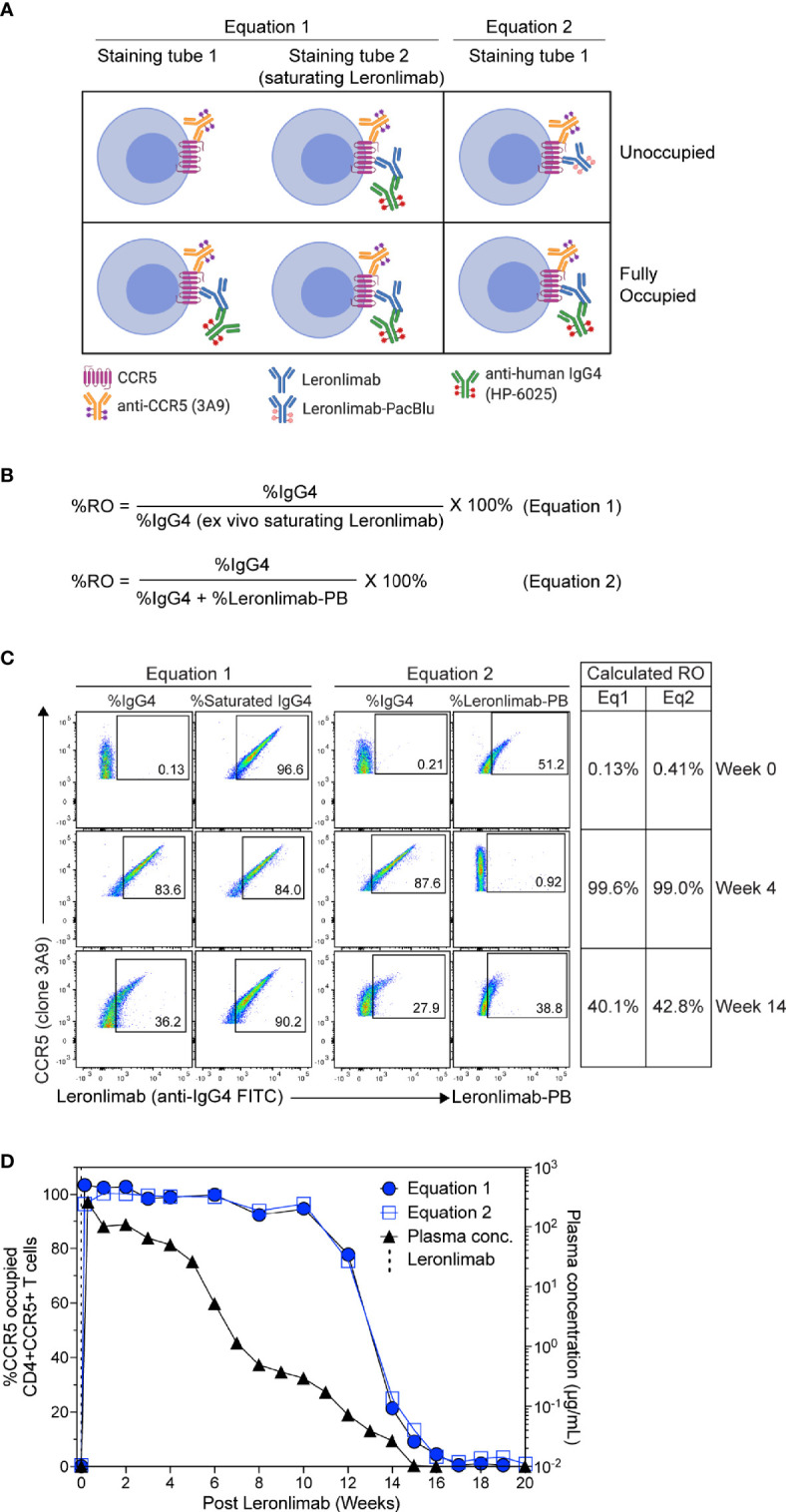
CCR5 receptor occupancy assay overview. **(A)** Flow cytometry diagram showing the interactions between anti-CCR5, Leronlimab, Leronlimab-PacBlu (PB), and anti-human IgG4 for the two equations using a CCR5 unoccupied (top) and fully occupied (bottom) scenario. **(B)** Equations for calculating CCR5 RO. **(C)** Representative flow cytometry plots displaying the different components needed to calculate for the two equations using a rhesus macaque that received a single 50 mg/kg SC Leronlimab injection. Equation 1 used %IgG4+ events within CD45+, singlet, live, CD3+, CD4+/CD8-, and CCR5+ events. Equation 2 used %IgG4+ and Leronlimab-PB+ events within CD45+, singlet, live, CD3+, CD4+/CD8-, and CCR5+ events. Table on the right shows the calculated CCR5 RO calculated by the two equations at study weeks 0, 4, and 14 post single Leronlimab injection. **(D)** Left Y-axis is for CCR5 RO by Leronlimab on peripheral blood CD4+CCR5+ T cells calculated by equation 1 (solid blue circle) and equation 2 (open blue square). Right Y-axis is for the longitudinal plasma concentration (solid black triangle) in blood samples from the treated macaque.

To measure CCR5 RO, we designed Leronlimab CCR5 RO assays based on methods previously established for RO measurements of anti-PD-1 antibodies in clinical trials (49, 50). The Leronlimab CCR5 RO assays consist of the three following critical components: 1) the CCR5-specific antibody clone 3A9 that does not compete with nor sterically hinder Leronlimab binding of CCR5 to track overall CCR5 expression, 2) the anti-human IgG4 antibody clone HP6025, which binds to the humanized IgG4 Fc of Leronlimab and 3) Pacific Blue-labeled Leronlimab (termed Leronlimab-PB), which binds to free CCR5 receptors not occupied by Leronlimab (**Figure 2A**). To measure the percentage of CCR5 RO on the surface of CD4+ T cells, two different methods were used as described in detail in the Materials and Methods section. CCR5 RO equation 1 measures Leronlimab-bound receptor by using the anti-human IgG4 antibody to measure Leronlimab-occupied CCR5 receptor directly (**Figure 2A**). CCR5 RO equation 2 measures unoccupied CCR5 receptors by using Pacific Blue-conjugated Leronlimab. In both methods, CCR5 staining with the 3A9 antibody is used to account for weekly variations in CCR5 expression prior to calculation of CCR5 RO (**Supplemental Figure 1**). CCR5 RO for Equation 1 is defined by the percentage of cells CCR5+ (measured by clone 3A9) and Leronlimab+ (measured by anti-human IgG4) divided by the percentage of cells CCR5+ and Leronlimab+ following incubation with 5 μg/mL, a saturating concentration of unlabeled Leronlimab (**Figures 2B, C**).


(*Equation* 1) $$\%RO=\frac{\%IgG4}{\%IgG4\;(ex\;vivo\;saturating\;Leronlimab)}\times 100\%$$

CCR5 RO for Equation 2 is defined as the percentage of cells CCR5+ (measured by clone 3A9) and Leronlimab+ (measured by anti-human IgG4) divided by the percentage of cells CCR5+ and Leronlimab+ (measured by the sum of anti-human IgG4 and Leronlimab-PB) cells following incubation with 5 μg/mL Leronlimab-PB.


(*Equation* 2) $$\%RO=\frac{\%IgG4}{\%IgG4+Leronlimab–PB}\times 100\%$$

Next, we tested the Leronlimab CCR5 RO assay using longitudinal peripheral blood samples from a rhesus macaque that received a single 50 mg/kg subcutaneous (SC) dose of Leronlimab. Representative flow cytometric plots and calculated CCR5 RO values using the two equations at three different study weeks are shown to demonstrate how the RO values are calculated (**Figure 2C**). We observed no CCR5 RO on peripheral blood CD4+ T cells immediately prior to Leronlimab dosing, followed by 100% CCR5 RO within eight hours following the SC injection (**Figure 2D**). CCR5 RO was then maintained at approximately 100% until the Leronlimab plasma concentration fell below 5 μg/mL at approximately six weeks post injection. Following this, CCR5 RO continually decreased to baseline level following complete washout of Leronlimab from plasma. Importantly, both RO methods yielded similar CCR5 RO measurements throughout the study and correlated with the Leronlimab plasma concentration. These results demonstrate the sensitivity and reproducibility of the CCR5 RO assay for monitoring Leronlimab RO *ex vivo.*

### Leronlimab Treatment Increases CCR5+CD4+ T Cell Counts in Healthy Rhesus Macaques

**Figure 3:**
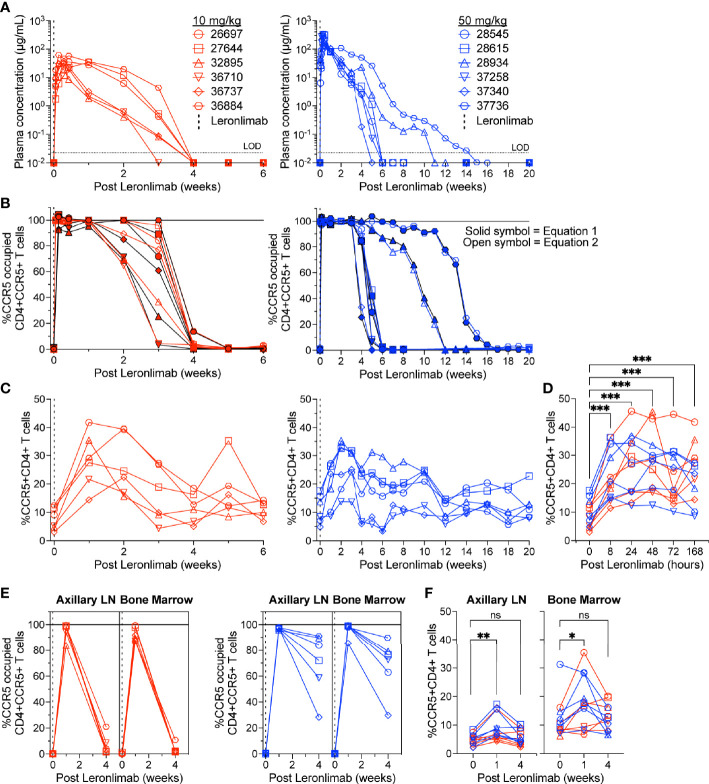
CCR5+CD4+ T cell frequencies increase with CCR5 RO. Leronlimab-naïve, SIV-naïve, rhesus macaques received a single 10 mg/kg (n=6; red; left) or 50 mg/kg (n=6; blue; right) SC Leronlimab. **(A)** Longitudinal Leronlimab concentration in plasma. **(B)** Longitudinal CCR5 RO by Leronlimab on peripheral blood CD4+CCR5+ T cells. **(C, D)** Peripheral blood CCR5+CD4+ T cell frequency for **(C)** longitudinal weekly timepoints, separated by treatment group and **(D)** hourly timepoints for both treatment groups within the first week post Leronlimab. **(E, F)** Axillary lymph node and bone marrow **(E)** CCR5 RO by Leronlimab and **(F)** %CCR5 on tissue-resident CD4+ T cells. **(B, E)** Solid symbol represents CCR5 RO calculated from equation 1 while open symbol represents CCR5 RO calculated from equation 2. P-values in panels **(D, F)** generated by Wilcoxon signed-ranked test; ns, not significant, *p < 0.05; **p < 0.01; ***p = 0.0005.

To further verify that CCR5 RO calculated by equation 1 and equation 2 were robust methods to longitudinally track Leronlimab CCR5 RO over time in blood and tissue, as well as to monitor the impact of CCR5 RO on CCR5+CD4+ T cell levels, we treated 12 rhesus macaques with a single 10 mg/kg or 50 mg/kg SC Leronlimab injection (n=6 per group). As expected, Leronlimab plasma levels peaked at lower levels and washed out more rapidly in macaques who received 10 mg/kg versus 50 mg/kg (**Figure 3A**). In both groups, however, Leronlimab treatment yielded full CCR5 RO on peripheral blood CD4+ T cells by eight hours post injection, and maintained >90% CCR5 RO for an average of 12.8 days and 32.6 days for the 10 mg/kg and 50 mg/kg groups, respectively (**Figure 3B**).

CCR5 antagonists impact expression of cell surface CCR5 and thus modulate the levels CCR5+CD4+ T cells circulating *in vivo*. Maraviroc treatment increased CCR5 expression on peripheral blood T cells in both humans and macaques, but the effect on CCR5 expression on tissue-resident T cells is unclear (42, 43, 51). In contrast, longitudinal treatment of macaques with the anti-CCR5 antibody HGS101 did not increase CCR5 expression, but rather decreased levels of CCR5+CD4+ T cells in both peripheral blood and lymph nodes (41). Therefore, we next sought to assess the impact of Leronlimab on CCR5+CD4+ T cells levels in both peripheral blood and within tissues. Following administration of Leronlimab at either dose we observed an increase in the frequencies of CCR5+CD4+ T cells circulating within peripheral blood that tracked with CCR5 RO (**Figure 3C**). In line with the rapid stabilization of CCR5 *in vitro* shown in **Figure 1**, statistically significant increases in peripheral blood CCR5+CD4+ T cell frequencies were found as early as eight hours post injection and maintained at every timepoint examined throughout the first week post injection (**Figure 3D**). Next, we examined the impact of Leronlimab on CCR5+CD4+ T cells from lymph node and bone marrow biopsies. Similar to peripheral blood results, we found no CCR5 RO on lymph node and bone marrow CD4+ T cells prior to Leronlimab injection, followed by high levels of CCR5 RO on CD4+ T cells from these tissues at one week post injection (**Figure 3E**). Reflective of plasma concentration and peripheral blood CCR5 RO results, levels of CCR5 RO by Leronlimab on tissue CD4+ T cells were very low at four weeks post injection in the 10 mg/kg treated group, while significant, but variable CCR5 RO levels remained on tissue CD4+ T cells from the 50 mg/kg treated group (**Figure 3E**). In line with these CCR5 RO values, we observed a statistically significant increase in frequencies of tissue CCR5+CD4+ T cells at one week post Leronlimab injection, concomitant with high levels of CCR5 RO on CD4+ T cells in those tissues (**Figure 3F**). Upon loss of CCR5 RO at week four post Leronlimab injection, the levels of CCR5+CD4+ T cells decreased and were no longer statistically different from pre-injection levels. These observations demonstrate that Leronlimab treatment increases CCR5+CD4+ T cell frequencies in both the peripheral blood and within lymphoid tissues, and that this phenomenon depends upon the degree of Leronlimab occupancy of CCR5.

### Leronlimab Treatment Suppresses SIVmac239 Replication Despite Increasing CCR5+CD4+ T Cell Levels

The observed increase in CCR5+CD4+ T cells in Leronlimab-treated macaques raised the possibility that Leronlimab could inadvertently exacerbate ongoing SIV infection by providing additional susceptible targets for viral replication. To study the impact of Leronlimab on CD4+ T cell dynamics and viral replication in an ongoing SIV infection, we treated a chronically SIVmac239-infected Mauritian cynomolgus macaque with weekly 50 mg/kg SC Leronlimab injections for 11 weeks.

**Figure 4:**
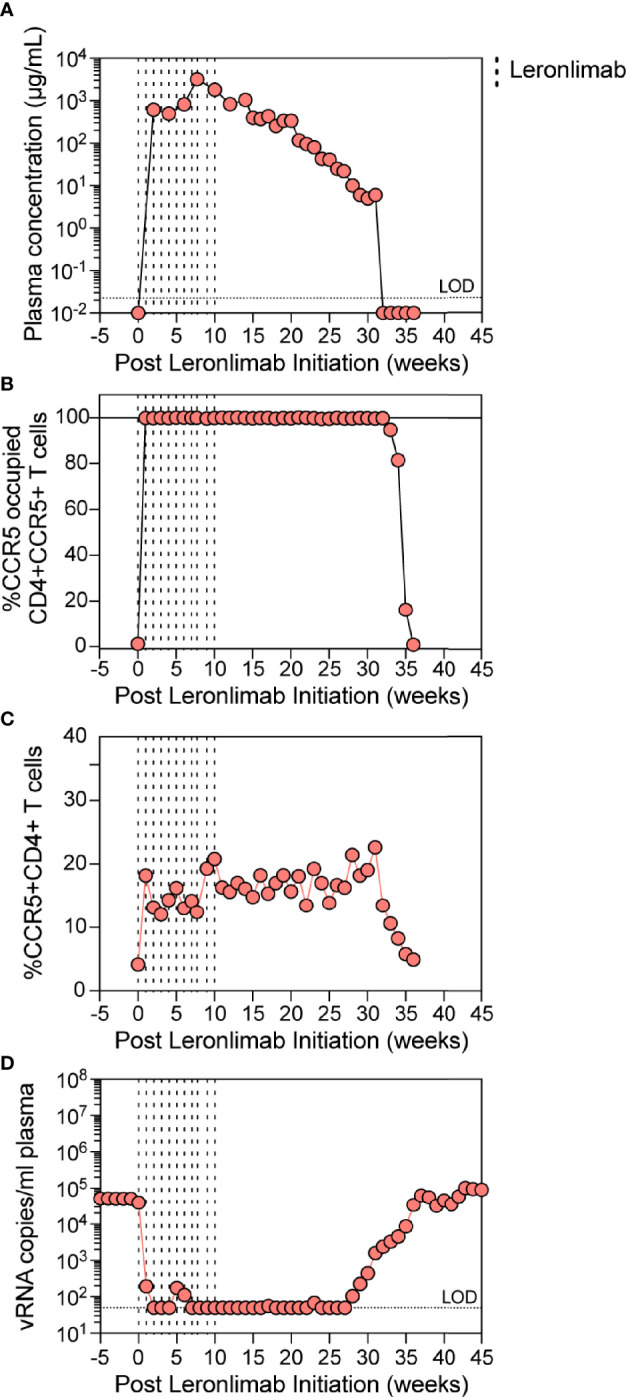
Leronlimab treatment of an SIVmac239-infected macaque. A cynomolgus macaque (n=1; 36484) was chronically infected with SIVmac239 prior to receiving weekly 50 mg/kg SC Leronlimab. **(A)** Longitudinal Leronlimab concentration in plasma. **(B)** Longitudinal CCR5 RO by Leronlimab on peripheral CD4+CCR5+ T cells. **(C)** Longitudinal peripheral blood CCR5+CD4+ T cell frequency. **(D)** Longitudinal SIV RNA copies per mL plasma. Vertical dashed lines represent each SC Leronlimab injection.

Similar to a single 50 mg/kg injection, complete CCR5 RO on peripheral blood CD4+ T cells was observed one week after the first dose and subsequently maintained for weeks after the final dose. However, compared to a single 50 mg/kg injection, repeated weekly 50 mg/kg injections for 11 weeks resulted in a longer duration of plasma Leronlimab, where complete Leronlimab plasma wash out and loss of CCR5 RO occurred at study weeks 32 and 36 post first dose, respectively (**Figures 4A** **, B**). As expected, based on our results above in SIV-naïve macaques, the frequency of CCR5+CD4+ T cells circulating in peripheral blood tracked with CCR5 RO, where it immediately increased after the first injection, began to decline with Leronlimab plasma washout, and returned to baseline level with loss of CCR5 RO (**Figure 4C**). Importantly, the increased frequencies of CCR5+CD4+ T cell targets did not exacerbate SIV replication. Instead, Leronlimab potently and completely suppressed SIV replication for approximately 20 weeks, during the time period where both full CCR5 RO and increased CCR5+CD4+ T cells were present in the blood (**Figure 4D**). As the Leronlimab plasma concentration declined and CCR5 RO was lost on CD4+ T cells, viral rebound occurred. SIVmac239 plasma viremia ultimately returned to pre-Leronlimab levels after complete loss of CCR5 RO. Therefore, the Leronlimab-induced increase in CCR5+CD4+ T cell targets did not exacerbate ongoing SIV replication; rather, the binding of Leronlimab to the CCR5 co-receptor used for viral entry protected these cells from infection and greatly diminished ongoing SIV replication, resulting in minimal plasma viremia during the period of complete CCR5 RO.

### Measurement of CCR5 RO in Leronlimab-Treated, HIV-Free Human Participants

**Figure 5:**
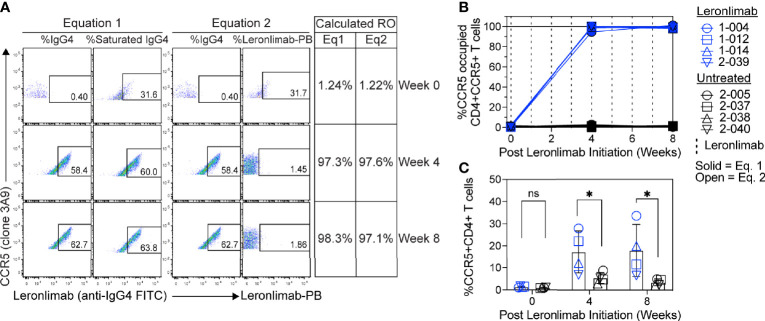
CCR5 RO assay on human samples. Blood samples were collected from human participants in ClinicalTrials.gov Identifier: NCT04678830 at study weeks 0, 4, and 8 post Leronlimab initiation. Leronlimab-treated (n=4; blue) participants received weekly 700 mg SC Leronlimab, while controls were untreated (n=4; black). **(A)** Representative flow cytometry plots showing the different components required to calculate CCR5 RO for equation 1 and 2. Table on the right shows the calculated RO at study weeks 0, 4, and 8. **(B)** Longitudinal CCR5 RO by Leronlimab on peripheral blood CD4+ T cells calculated with equation 1 (solid blue) and equation 2 (open blue). **(C)** Longitudinal CCR5+CD4+ frequency in peripheral blood. Vertical dashed line represents each SC Leronlimab injection. P-values in panel **(C)** calculated by two-way repeated measures ANOVA with Sidak’s correction for multiple comparisons; ns, not significant; *p < 0.05.

Currently, Leronlimab is undergoing testing in clinical trials for both HIV and HIV-unrelated indications. With the successful demonstration of the CCR5 RO assay in non-human primate models, we next sought to extend the pre-clinical CCR5 RO technique for clinical applications. While the majority of the antibodies utilized in the macaque-specific assay are human-specific antibodies that cross react with the macaque orthologue, we adapted and refined the antibody clones utilized for optimal staining on human cells (**Supplemental Table 2**). To determine the performance of the clinical-grade CCR5 RO assay, we obtained blinded clinical samples from eight participants enrolled in a phase 2, two-arm, randomized, double blind, placebo-controlled study on the effects of Leronlimab treatment in long COVID-19 (ClinicalTrials.gov Identifier: NCT04678830). Enrolled participants were randomized to receive either weekly 700 mg SC Leronlimab injections or placebo throughout an eight-week study period, with a total of three clinical visits at weeks 0, 4, and 8 after the first Leronlimab initiation. As in our pre-clinical study models, the two equations calculated comparable CCR5 RO percentages from all three clinical visits in Leronlimab-treated human participants (**Figure 5A**). Furthermore, the CCR5 RO assay correctly identified individuals treated with Leronlimab versus placebo following unblinding, as treated individuals presented at baseline with no CCR5 RO, but then achieved full CCR5 RO on peripheral blood CD4+ T cells at the two follow-up clinical visits, while placebo-treated participants never presented with CCR5 RO (**Figure 5B**). In line with the preclinical macaque results, we observed increased frequencies of circulating CCR5+CD4+ T cells following Leronlimab dosing. After Leronlimab initiation, frequencies of CCR5+CD4+ T cells circulating in the peripheral blood in Leronlimab-treated participants were statistically higher than in untreated participants (**Figure 5C**). Thus, the longitudinal CD4+ T cell CCR5 RO in Leronlimab-treated participants and lack of measurable CCR5 RO in Leronlimab-untreated participants demonstrates the robustness and sensitivity of the clinical CCR5 RO assay. Indeed, in both macaques and humans, CCR5 RO in the absence or presence of saturating plasma concentrations of Leronlimab was approximately 1% and 99%, respectively (**Table 1**). Cumulatively, these results establish precise methods to measure CCR5 RO in a pre-clinical nonhuman primate species and in human participants in clinical trials, and demonstrate that Leronlimab CCR5 RO induces increased frequencies of CCR5+CD4+ T cells.

**Table 1:** Calculated CCR5 RO by Leronlimab on peripheral blood CD4+ T cells from Leronlimab-treated or untreated humans and macaques.

Leronlimab condition	Species	Mean % CCR5 RO (SD, N)	
		Equation 1	Equation 2
Untreated	Human	1.02% (0.62, 8)	0.65% (0.52, 8)
	Macaque	0.38% (0.41, 12)	0.08% (1.06, 12)
Treated	Human	98.39% (2.47,4)	99.73% (0.57, 4)
	Macaque	100.83% (4.33, 12)	99.84% (0.59, 12)

## Discussion

Here, we created and validated two different methods of calculating CCR5 RO by the anti-CCR5 antibody Leronlimab. The methods generated comparable longitudinally CCR5 RO percentages in rhesus macaques that received a single 10 mg/kg or 50 mg/kg SC Leronlimab injection. Additionally, both methods were highly sensitive, with baseline values of 1% and fully saturated values of 99% when tested in human and non-human primates. These results are in contrast to the commonly used MIP-1β internalization assay utilized for Maraviroc, which is associated with background levels of approximately 25% in human samples (38) and yields values in excess of 100% in maraviroc-treated and -untreated rhesus macaques (39). Higher RO percentages calculated by the MIP-1β internalization assay may be due to fluctuating CCR5 frequencies or incomplete CCR5 internalization upon MIP-1β binding. In contrast, our methods did not depend on receptor internalization and all mathematical components used were gated on CCR5+ cells, compensating for any fluctuation in CCR5 frequency and allowing for precise calculation of RO. Finally, the pre-clinical Leronlimab CCR5 RO assay was extended into human participants, demonstrating the ability to longitudinally and robustly monitor CCR5 RO.

Similar to maraviroc, we found that Leronlimab stabilized surface CCR5 molecules and prevented its internalization following ligand binding. Indeed, this shared feature of both drugs likely explains their shared ability to increase frequencies of CCR5+CD4+ T cells in both humans and macaques. Because CCR5+CD4+ T cells are target cells for HIV/SIV infection, increasing the frequencies of susceptible cells could raise concerns of increased HIV/SIV replication. However, we found that weekly Leronlimab treatment in a chronically SIVmac239-infected macaque fully suppressed plasma viremia for over 20 weeks despite a rise in the CCR5+CD4+ T cell frequency immediately after the first Leronlimab injection. Both suppression of viral replication and increased CCR5+CD4+ T cell levels were temporally associated with full CCR5 RO on peripheral blood CD4+ T cells, underscoring the need to measure CCR5 RO in studies utilizing CCR5-blocking agents.

Because CCR5 is involved in multiple pathophysiologic processes, Leronlimab is being explored in clinical trials for both HIV and non-HIV indications. In HIV-positive participants, Leronlimab suppressed plasma viremia after a single 5 or 10 mg/kg SC Leronlimab injection, while Leronlimab monotherapy contributed to the maintenance of viral suppression for over six years (52–55). Moreover, the only two cases of HIV cure occurred after hematopoietic stem cell transplantation (HSCT) with donor cells homozygous for CCR5Δ32 (56, 57), while similar studies using donor cells that were wild-type CCR5 or heterozygous CCR5Δ32 led to eventual viral rebound (58). For this reason, there is intense focus on genetically disrupting *ccr5* to abolish cell surface CCR5 expression in HIV-positive individuals (59, 60). However, CCR5 has many protective roles, making it difficult to predict the long-term biological effects or consequences of permanently disrupting *ccr5* expression in humans. A logical approach to reproduce the phenotypic protection seen in homozygous CCR5Δ32 individuals is to instead use CCR5-blocking pharmacologic agents such as Leronlimab. Moreover, the therapeutic use of Leronlimab extends beyond HIV treatment due to its diverse roles. CCR5 is expressed in over 95% of triple-negative breast cancers (61) and influences breast cancer progression (9). In a murine model, Leronlimab prevented and reduced breast cancer metastasis suggesting a role for Leronlimab in the treatment of neoplasia (62). As CCR5 is central in inflammatory immune responses, it is currently being studied as a therapeutic for severe and critical SARS-CoV-2 infections (44, 63–65) and graft-versus-host disease (GVHD), where Leronlimab treatment reduced xeno-GVHD after HSCT of human cells to mice (66). Finally, Leronlimab is currently in phase 1 and 2 clinical studies to treat metastatic colorectal cancer, nonalcoholic Steatohepatitis, and long COVID after SARS-CoV-2 infection, demonstrating the diverse applicability of this safe and effective CCR5-targeting agent.

The appeal of monoclonal antibody-based therapeutic agents is growing due to their longer half-life and promising safety profile, and this is reflected in the dramatic rise in FDA approvals and commercial use of antibody treatments in recent years (67, 68). The CCR5 RO assays described here will be an important study measurement for any CCR5 antibody-based agent, and can be modified for any antibody-based agents that have a clearly defined cell surface protein target. In the case of the anti-CCR5 antibody Leronlimab, the ability to accurately measure CCR5 RO will advance both pre-clinical and clinical studies, furthering our understanding of the immunological impacts of CCR5 for multiple pathophysiologic processes.

## Data Availability Statement

The original contributions presented in the study are included in the article/**Supplementary Material**. Further inquiries can be directed to the corresponding authors.

## Ethics Statement

The protocol was approved by the institutional review board at Amarex Clinical Research, Arthritis and Rheumatic Disease Specialties, and Center for Advanced Research and Education (CARE). ClinicalTrials.gov identifier (NCT number), NCT04678830. The patients/participants provided their written informed consent to participate in this study. The animal study was reviewed and approved by Oregon National Primate Research Center (ONPRC) Institutional Animal Care and Use Committee (IACUC).

## Author Contributions

JBS conceptualized the studies and experiments. XC and HW managed macaque studies, assisted by JR, and JH. CR ran the clinical trial, while CH, AS, DM, KB, and SH performed analysis. MF, RB, and JS managed the animal care and procedures. XC, HW, GW, MT, CH, JR, JH, CW, CB, CP, AS, DM, KB, SK, NP, CR, SH, and JBS investigated. XC and JBS designed and validated CCR5 RO assays in macaques, while JBS and SH adapted assay for human clinical use. XC and JBS drafted the manuscript, and all authors contributed to editing. JBS, SH, SK, and NP acquired funding. All authors contributed to the article and approved the submitted version.

## Funding

This work is supported by National Institute of Allergy and Infectious Diseases (NIAID) grants R01 AI129703, R01 AI54559, and R21 AI54559 to JBS, and the Oregon National Primate Research Center Core grant P51 OD011092 from the National Institutes of Health, Office of the Director.

## Conflict of Interest

Authors NP, SK and CPR were employees of company CytoDyn, owner and developer of Leronlimab. JBS, SH, HLW, and GMW have received compensation for consulting for CytoDyn, a company that may have commercial interests in the results of this research. JBS has also received compensation for serving on the scientific advisory board of CytoDyn. This potential conflict of interest has been reviewed and managed by the Oregon Health & Science University.

The remaining authors declare that the research was conducted in the absence of any commercial or financial relationships that could be construed as a potential conflict of interest.

## Publisher’s Note

All claims expressed in this article are solely those of the authors and do not necessarily represent those of their affiliated organizations, or those of the publisher, the editors and the reviewers. Any product that may be evaluated in this article, or claim that may be made by its manufacturer, is not guaranteed or endorsed by the publisher.
