# Supplementary material for: CCR5 Receptor Occupancy Analysis Reveals Increased Peripheral Blood CCR5+CD4+ T Cells Following Treatment With the Anti-CCR5 Antibody Leronlimab
Source: Front Immunol. 2021 Nov 19;12:794638. doi: 10.3389/fimmu.2021.794638 (PMC8640501; doi:10.3389/fimmu.2021.794638)
Supplement: Supplementary file 1 [file DataSheet_1.docx]

Supplementary Material

CCR5 receptor occupancy analysis reveals increased peripheral blood CCR5+CD4+ T cells following treatment with the anti-CCR5 antibody Leronlimab.

Xiao L. Chang^1^, Helen L. Wu^1^, Gabriela M. Webb^1^, Meenakshi Tiwary^1^, Colette Hughes^1^, Jason S. Reed^1^, Joseph Hwang^1^, Courtney Waytashek^1^, Carla Boyle^1^, Cleiton Pessoa^1^, Andrew W. Sylwester^1^, David Morrow^1^, Karina Taylor^1^, Miranda Fischer^2^, Scott Kelly^3^, Nader Pourhassan^3^, Rachele M. Bochart^2^, Jeremy Smedley^2^, Christopher P. Recknor^3^, Scott G. Hansen^1*^, Jonah B. Sacha^1,2*^


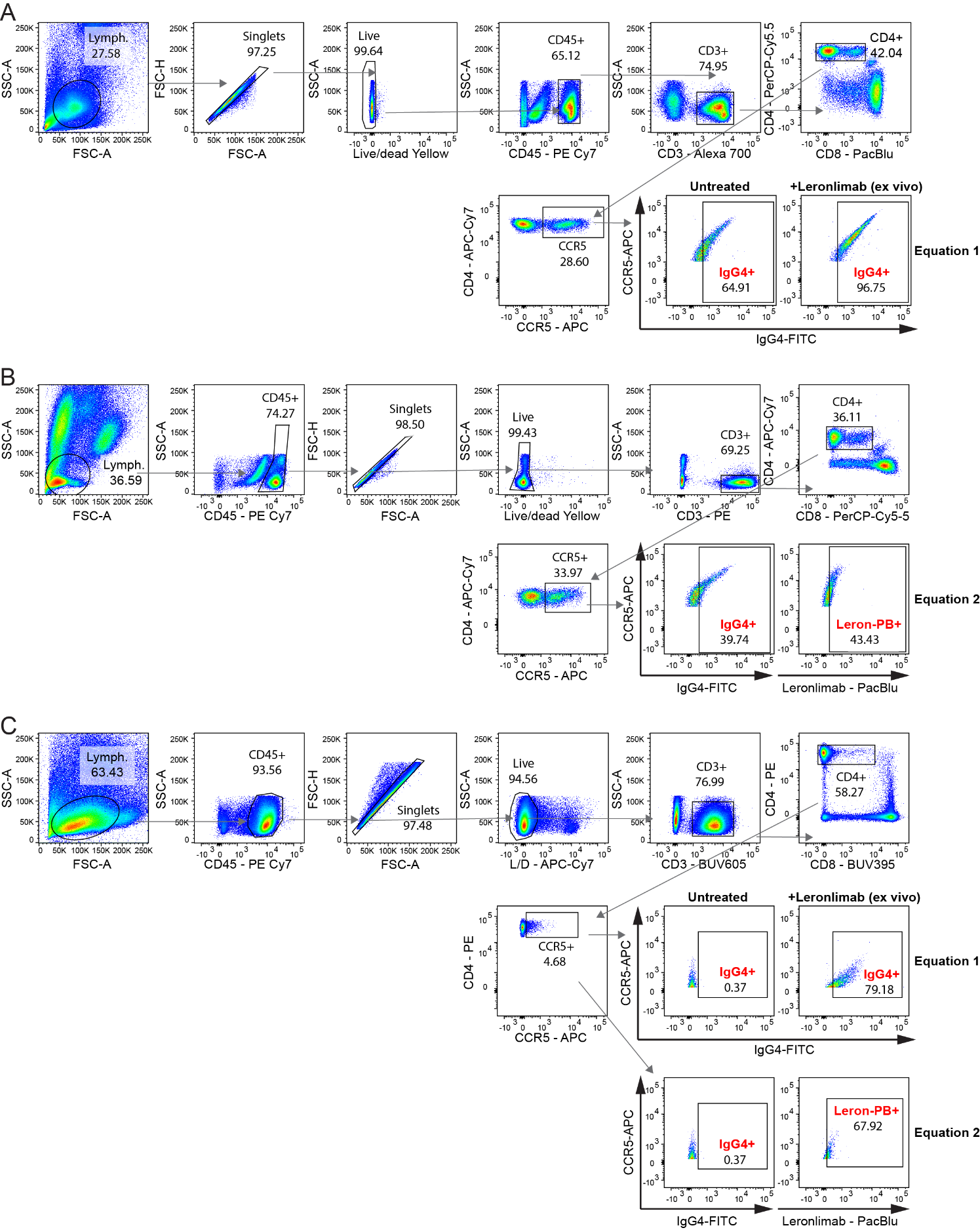


**Supplemental Figure 1.** Representative flow cytometry gating strategies for CCR5 RO using **(A)** equation 1 and **(B)** equation 2 in partially-occupied macaques, and **(C)** equation 1 and 2 in unoccupied human.

| CCR5 RO | Species | Tubes | Leronlimab | Leronlimab-PB | Anti-IgG4 FITC | Anti-CCR5 APC |
| --- | --- | --- | --- | --- | --- | --- |
| Equation 1 | Macaque | 1 | - | - | - | - |
|  |  | 2 | - | - | **+** | **+** |
|  |  | 3 | 5 μg/mL | - | **+** | **+** |
| Equation 2 | Macaque | A | - | - | **-** | **-** |
|  |  | B | - | - | - | **+** |
|  |  | C | - | 5 μg/mL | **+** | **+** |
| Combined equations 1 and 2 | Human | H1 | - | - | - | - |
|  |  | H2 | - | - | - | **+** |
|  |  | H3 | - | 5 μg/mL | **+** | **+** |
|  |  | H4 | 5 μg/mL | - | **+** | **+** |

**Supplemental Table 1.** Staining tubes for CCR5 RO equation 1 and equation 2 used in macaque and human analysis.

| *BD LSR II* | | | **Macaque Equation 1** | | **Macaque Equation 2** | |
| --- | --- | --- | --- | --- | --- | --- |
| Laser | Filter | Fluorophore Detector | Pre-Surface | Surface | Pre-Surface | Surface |
| - | - | - | LRM-pure |  | LRM-pure |  |
| - | - | - |  |  |  |  |
| Violet | 450/50 | PacBlue |  | CD8α (RPA-T8) |  | LRM-PB |
| Violet | 525/50 | BV510 |  |  |  |  |
| Violet | 560/40 | QD655 |  |  |  |  |
| Violet | 585/42 | BV570 |  |  |  |  |
| Violet | 605/40 | BV605 |  | L/D |  | L/D |
| Violet | 660/40 | BV650 |  |  |  |  |
| Violet | 705/70 | BV711 |  |  |  |  |
| Violet | 780/60 | BV786 |  |  |  |  |
| Blue | 515/20 | FITC | IgG4 (HP-6025) |  | IgG4 (HP-6025) |  |
| Blue | 710/50 | PerCP Cy5.5 |  | CD4 (L200) |  | CD8 (SK1) |
| Green | 575/25 | PE |  | CD95 (DX2) |  | CD3 (SP34-2) |
| Green | 610/20 | PE CF594 |  | CD28 (CD28.2) |  | CD14 (RMO52) |
| Green | 660/40 | PE Cy5 |  |  |  |  |
| Green | 710/50 | PE Cy5.5 |  |  |  |  |
| Green | 780/40 | PE Cy7 |  | CD45  (D058-1283) |  | CD45 (D05-1283) |
| Red | 660/20 | APC |  | CCR5  (3A9) |  | CCR5 (3A9) |
| Red | 710/50 | A700 |  | CD16 (SP34-2) |  | CD16 (3G8) |
| Red | 780/60 | APC-Cy7 |  |  |  | CD4 (OKT4) |

**Supplemental Table 2**: Antibody table for BD LSR II. Antibodies used in the CCR5 RO staining in macaque and human, with antibody clone names listed in parentheses. LRM-Pure = unconjugated, parental Leronlimab; LRM-PB = Leronlimab-Pacific Blue; L/D = live/dead.

| *BD FACSymphony* | | | **Human RO** | |
| --- | --- | --- | --- | --- |
| Laser | Filter | Fluorophore Detector | Pre-Surface | Surface |
| - | - | - | LRM-Pure |  |
| - | - | - |  |  |
| UV | 378/29 | BUV395 |  | CD8α. (RPA-T8) |
| UV | 515/30 | BUV496 |  | CD16 (3G8) |
| UV | 740/35 | BUV737 |  | CD95 (DX2) |
| Violet | 450/50 | PacBlue |  | LRM-PB |
| Violet | 525/50 | BV510 |  |  |
| Violet | 586/15 | BV570 |  |  |
| Violet | 610/20 | BV605 |  | CD3 (UCHT1) |
| Violet | 670/30 | BV650 |  |  |
| Violet | 710/50 | BV711 |  | CD14 (M5E2) |
| Violet | 780/60 | BV786 |  |  |
| Blue | 530/30 | FITC | IgG4 (HP-6025) |  |
| Blue | 575/25 | PE |  | CD4 (SK2) |
| Blue | 610/20 | PE CF594 |  | CD28 (CD28.2) |
| Blue | 710/50 | PerCP Cy5.5 |  |  |
| Blue | 780/60 | PE-Cy7 |  | CD45 (2D1) |
| Red | 670/30 | APC |  | CCR5 (3A9) |
| Red | 710/50 | A700 |  |  |
| Red | 780/60 | APC-Cy7 | L/D |  |

**Supplemental Table 3**: Antibody table for BD FACSymphony. Antibodies used in the CCR5 RO staining in macaques and human, with antibody clone names listed in parentheses. LRM-Pure = unconjugated, parental Leronlimab; LRM-PB = Leronlimab-Pacific Blue; L/D = live/dead.
